# Alterations of default mode functional connectivity in individuals with end-stage renal disease and mild cognitive impairment

**DOI:** 10.1186/s12882-019-1435-6

**Published:** 2019-07-05

**Authors:** Haitao Lu, Zhengzhang Gu, Wei Xing, Shanhua Han, Jiangfen Wu, Hua Zhou, Jiule Ding, Jinggang Zhang

**Affiliations:** 1 Department of Radiology, The Third Affiliated Hospital of Soochow University, Changzhou, China; 2 Department of Radiology, Shanghai Fourth People’s Hospital, Shanghai, China; 3 GE Healthcare, Shanghai, China; 4 Department of Nephrology, The Third Affiliated Hospital of Soochow University, Changzhou, China

**Keywords:** Resting state fMRI, End stage renal disease, Mild cognitive impairment

## Abstract

**Background:**

Mild cognitive impairment (MCI) occurs frequently in many end stage renal disease (ESRD) patients, may significantly worsen survival odds and prognosis. However, the exact neuropathological mechanisms of MCI combined with ESRD are not fully clear. This study examined functional connectivity (FC) alterations of the default-mode network (DMN) in individuals with ESRD and MCI.

**Methods:**

Twenty–four individuals with ESRD identified as MCI patients were included in this study; of these, 19 and 5 underwent hemodialysis (HD) and peritoneal dialysis (PD), respectively. Another group of 25 age-, sex- and education level-matched subjects were recruited as the control group. All participants underwent resting-state functional MRI and neuropsychological tests; the ESRD group underwent additional laboratory testing. Independent component analysis (ICA) was used for DMN characterization. With functional connectivity maps of the DMN derived individually, group comparison was performed with voxel-wise independent samples t-test, and connectivity changes were correlated with neuropsychological and clinical variables.

**Results:**

Compared with the control group, significantly decreased functional connectivity of the DMN was observed in the posterior cingulate cortex (PCC) and precuneus (Pcu), as well as in the medial prefrontal cortex (MPFC) in the ESRD group. Functional connectivity reductions in the MPFC and PCC/Pcu were positively correlated with hemoglobin levels. In addition, functional connectivity reduction in the MPFC showed positive correlation with Montreal Cognitive Assessment (MoCA) score.

**Conclusion:**

Decreased functional connectivity in the DMN may be associated with neuropathological mechanisms involved in ESRD and MCI.

## Background

End stage renal disease (ESRD) correspond to stage 5 chronic kidney disease (CKD), when chronic renal failure has progressed to the point that the kidneys are permanently functioning at less than 10% of normal capacity [1]. In additional to renal failure, mild cognitive impairment (MCI) is a common comorbidity in ESRD cases [2]. This cognitive problem in ESRD could be caused by uremia, thiamine deficiency, hypertension, dialysis, transplant rejection and/or electrolyte disturbances [3]. Patients with MCI are of high risk of developing dementia, which may significantly worsen survival odds and prognosis [4]. However, the exact neuropathological mechanisms of MCI combined with ESRD remain unclear, thus hampering the development of efficient treatments.

Conventional medical imaging has played an important role in exploring the structural and functional brain mechanisms associated with ESRD, such as cerebral infarction, intracranial hemorrhage, subcortical white matter lesions, volume reduction, and metabolic disturbances [5–7]. However, due to the limitation of insensitivity to early morphological changes, existing imaging approaches in the clinical setting may be improved by additional assessment of functional networks.

Recently, much attention has been paid to resting state functional magnetic resonance imaging (rsfMRI) [8, 9], which constitutes a novel paradigm that examines spontaneous brain function by using blood oxygen level–dependent contrast in the absence of a task. Changes of blood oxygen saturation in some brain regions indicate functional alterations in these areas. Therefore, changes of functional connectivity in rsfMRI could reflect the functional status of the brain. Alterations in resting state networks have been identified in many diseases, such as Alzheimer’s disease [10], traumatic brain injury [11], hepatic encephalopathy [12], and ESRD [13–16]. In these studies, abnormal functional connectivity of the default mode network (DMN) has attracted great attention. The DMN is one of the resting state functional networks, which contain meaningful information related to spontaneous brain activity [10] and may be affected by neurological complications related to ESRD. Luo et al. [15] reported that ESRD patients show lower ALFF values in DMN regions compared with healthy control subjects. Ma et al. [14] and Qiu et al. [16] found that ESRD patients exhibit significantly decreased functional connectivity in several regions of the DMN by seed-based analysis.

In contrast to seed-based analysis, which heavily depends on a priori seeds defined, independent component analysis (ICA) requires no prior knowledge of a network, and hence may provide complementary/confirmatory information [9]. Using ICA, Ni et al. [13] extracted the DMN component in ESRD patients successfully, and found that the patients exhibit significantly decreased functional connectivity in the PCC, precuneus, and MPFC compared with control subjects, with functional connectivity of the MPFC positively correlated with digital symbol test score.

These findings of DMN alterations associated with ESRD are insightful; however, previous studies could not perform elaborate grouping according to the degree of cognitive impairment, and did not rule out the effects of comorbidities, such as diabetes and cardiovascular disease, in conclusions made about ESRD. In the present study, we specifically excluded ESRD patients complicated with diabetes mellitus, and used ICA to investigate FC alterations of the DMN. Focusing on ESRD patients complicated with MCI, we also examined potential associations of FC changes of the DMN with neuropsychological and clinical variables.

## Methods

### Subjects

This prospective study was approved by the Medical Research Ethics Committee of our hospital, and written informed consent from each subject was obtained before the study. From April 2016 to June 2017, 31 individuals (including outpatients and inpatients; all right-handed) with ESRD combined with MCI were initially recruited in this study. For inpatients, the cause of hospitalization were concurrent diseases, such as lung infection and hypertension, which may not impact the functional status of the brain. Primary diseases in the ESRD group included chronic glomerulonephritis (*n* = 7), polycystic kidney (*n* = 4), IgA nephropathy (*n* = 2), nephrotic syndrome (*n* = 2), hypertension (*n* = 1), and unknown ailments (*n* = 8). MCI was diagnosed according to Petersen criteria [17] for amnestic MCI: 1) memory complaint corroborated by an informant, 2) objective memory impairment for age, education and gender, 3) essentially preserved general cognitive function, 4) largely intact functional activities, 5) no dementia. For criterion 2), the Clinical Dementia Rating scale (CDR) [18] was used to identify MCI. CDR scores were obtained via a semi-structured interview of patients and informants, and the subject’s cognitive status was rated in six domains of functioning, including memory, orientation, judgment and problem solving, community affairs, home and hobbies, and personal care. Performances in these domains were assessed by a clinician with 9 years of experience in nephrology (HZ). When a subject had an overall CDR index of 0.5, the diagnosis of MCI was established. All subjects completed laboratory tests (serum creatinine, blood urea nitrogen and hemoglobin) within 72 h before MRI for evaluating renal function and the physical status. Dialysis modality and duration were obtained from the patient’s medical history. Neuropsychological tests, including Mini-Mental State Examination (MMSE) [19] and MoCA [20], were also completed within one hour before MR imaging to quantify objective memory impairment and essentially preserved general cognitive function of ESRD cases.

Exclusion criteria were: (a) a history of psychiatric disorders; (b) major neurologic disorders such as severe traumatic brain injury, stroke, epilepsy and tumor); (c) drug or alcohol abuse; (d) a history of diabetes; and (e) head motion more than 3.0 mm or 3.0° during fMRI. Based on the above criteria, 7 participants were excluded for cerebral hematoma (*n* = 2), a history of traumatic injury (*n* = 1) and excessive head motion (*n* = 4), resulting in a study sample of 24 participants (11 men and 13 women; mean age of 50.58 ± 9.49, ranging from 31 to 65 years). In this sample, 19 and 5 participants underwent hemodialysis (HD) and peritoneal dialysis (PD), respectively.

Twenty-five matching controls in terms of age, sex ratio and education level were recruited from the local community. Self-report of good health with normal renal function, confirmed by laboratory data from community doctors within 12 months of testing, no history of psychiatric or neurologic disease, and normal-abdominal ultrasonographic (US) imaging. All participants were right-handed. The control group completed the same procedure of MRI and neuropsychological tests within one hour before MRI imaging.

### MR imaging data acquisition

A 3.0 Tesla MR scanner (Achieva, Philips, Best, the Netherlands) and a 16-channel phased array head coil were used to acquire all MRI data. During MRI scans, participants were instructed to lie quietly with eyes closed but staying awake. A foam pad was used to reduce head motion. The degree of patient cooperation was verified after the examination. Conventional imaging sequences, including axial T1- and sagittal T1-weighted images and coronal T2 fluid-attenuated inversion-recovery images, were obtained for every subject to detect clinically silent lesions. For co-registration and normalization of resting-state functional MR imaging data, high-resolution T1-weighted 3D anatomical images were obtained in sagittal slices using a magnetization-prepared rapid gradient-echo sequence (TR/TE, 8.0/3.9 ms; slice thickness, 1 mm; 160 slices; flip angle, 8°; field of view, 250 × 250 mm^2^; acquisition matrix, 252 × 227; voxel size in acquisition, 0.99 × 1.10 × 2.00 mm^3^; voxel size after reconstruction, 0.98 × 0.98 × 1.00 mm^3^). Functional data were collected by a gradient-echo echo-planar sequence in axial slices parallel to the line through the anterior and posterior commissures: TR/TE, 2000/30 ms; slice thickness, 4 mm; gap between slices, 0.4 mm; field of view, 240 × 240 mm^2^; acquisition matrix, 120 × 117; resolution, 2.00 × 2.05 × 4.00 mm^3^; flip angle, 90°; fold-over direction, AP; EPI factor, 41. Each rsfMRI sequence contained 250 volumes with 30 slices.

### Data preprocessing

Data were preprocessed with statistical parametric mapping (SPM8, *http://www.fil.ion.ucl.ac.uk/spm/*) in Matlab (MathWorks, Natick, Mass). First, 3D anatomical data were reoriented, skull stripped, and segmented into gray matter, white matter and cerebrospinal fluid regions. Then, the first 10 volumes of rsfMRI data were discarded for magnetization equilibrium and participant adaptation. Slice timing and head motion corrections were performed subsequently followed by T1-EPI co-registration. Afterwards, spatial normalization was performed based on the unified segmentation to the T1 image and the fMRI data was warped into the standard space of the Montreal Neurologic Institute (MNI) template with a resampled voxel size of 3 × 3 × 3 mm^3^. Finally, images were spatially smoothed by convolution with an isotropic Gaussian kernel of FWHM = 8 mm.

### Independent component analysis

Group ICA was performed with the software package of GIFT (Vision 2.0e; *http://icatb.sourceforge.net/*). GIFT linearly decomposes 4D rsfMRI data into spatially independent sources, each of which is considered an intrinsically connected brain network [21]. To determine the number of independent components, dimension estimation was carried out individually using the minimum description length criterion [22], and averagely 21 components were obtained. This averaged independent component was used for each subject for independent component analysis separation. Then, functional MR imaging data of all subjects in both groups were concatenated in the temporal dimension, and the data set was separated into independent components by the InfoMax algorithm. After the ICA, a DMN template described in previous study [23] was used to select the best fit of the remaining low-frequency components in each subject. The template-matching procedure was realized by subtracting the average IC *z* score of voxels in the template from that outside the template, and the component with the maximum difference (goodness of fit) was selected [13]. The z scores used here reflected the degree to which a given voxel’s time series correlated with the time series corresponding to a specific independent component, scaled by the standard deviation of the error term. Therefore, the z score could be used to reflect the amplitude of regional activity relative to the background noise.

In this study, component 3 had the maximum difference (0.323), but followed closely by component 15 (0.274), so both were selected for subsequent group comparisons. The IC map of component 3 predominantly consisted of the posterior cingulate cortex (PCC), precuneus, bilateral inferior parietal and medial prefrontal cortex (MPFC), which was similar to a posterior-DMN. The IC map of component 15 was predominantly composed of the MPFC, PCC, bilateral inferior parietal, which was similar to an anterior-DMN [24].

### Statistical analysis

With SPM8, statistical analysis for rsfMRI data was performed by one sample (within group) and independent-samples (group contrast) t-tests. A voxel-wise threshold of *P* < 0.001 was applied, and false positives from multiple comparisons were controlled by the Alphasim procedure (Alphasim; *http://afni.nih.gov/afni/docpdf/AlphaSim.pdf*), with a cluster size greater than 22 voxels to remove false-positives and maintain true-positive sensitivity. For each component, within-group clusters of control and ESRD patients were combined with a logical “AND” operation, and subsequent group contrast was only considered in this conjunction mask. In two-sample t-tests, age and sex were included as covariates, and the voxel-wise threshold was set at *P* < 0.05. The result was corrected for multiple comparison with cluster sizes greater than 428 voxels (component 3) and 325 voxels (component 15). These cluster thresholds were determined by a Monte Carlo simulation implemented in the DPABI software package (*http://rfmri.org/DPABI*) with parameters of *P* < 0.05/voxel and 3D Gaussian smoothness of FWHM = 8 mm [25].

Statistical analysis for demographic and clinical data was performed by SPSS (version 16.0; SPSS, Chicago, III). Significant regions in group differences of components 3 and 15 were considered regions of interest (ROIs) [26] for individual IC z scores to be extracted from. Pearson correlation was used to assess associations of average IC *z* score of each participant in the ROI with clinical variables/psychological parameters (serum creatinine, blood urea nitrogen, hemoglobin, MMSE and MoCA scores) in the patient group.

### Reproducibility analysis

A split-half analysis was used to assess result reproducibility. The control and ESRD groups were both divided into two age- and gender- matched subgroups: control-1 (13 participants; 5M8F; age, 46.23 ± 12.73 years), control-2 (12 participants; 4M8F; age, 46.42 ± 11.74 years), ESRD-1 (12 participants; 5M7F; age, 49.92 ± 9.95 years) and ESRD-2 (12 participants; 6M6F; age, 51.17 ± 9.59 years). For each significant region identified in whole-sample group comparison, we extracted IC z scores from the ROIs and performed group comparison again across different subgroups. If differences between subgroups of the same diagnosis (e.g. control-1 vs. control-2) were insignificant but those between subgroups of different diagnoses (e.g. control-1 vs. ESRD-1) significant, the whole-sample results were considered to be reproducible.

## Results

The demographic and clinical data of all participants are summarized in Table 1. While group differences in age (*P* = 0.173), gender (*P* = 0.484), and education level (*P* = 0.386) were non-significant, the ESRD group had significantly lower MMSE (*P* = 0.01) and MoCA scores (*P* < 0.001). The duration of dialysis for individuals with ESRD was more than 3 months. Blood urea nitrogen and serum creatinine levels were out of respective normal ranges.

**Table 1:** Demographic and Clinical Data of all participants

Variables	ESRD group (*n* = 24)	Control group (*n* = 25)	*P* Value
Gender (M/F)	11/13	9/16	0.484^a^
Age (years)	50.58 ± 9.49 (31–65)	46.28 ± 12.07 (28–65)	0.173^b^
Education(years)	11.21 ± 3.16 (4–16)	12.2 ± 4.66 (3–19)	0.386^b^
MMSE	27.75 ± 1.75 (24–30)	28.88 ± 1.17 (27–30)	0.01^b^
MoCA	22.62 ± 2.10 (17–25)	27.8 ± 1.26 (26–30)	< 0.001^b^
Dialysis modality (PD/HD)	5/19		
Dialysis duration (months)	24.46 ± 16.55 (3–168)		
Hemoglobin (g/L)	101.17 ± 12.39 (77–122)		
Blood urea nitrogen (mmol/L)	22.85 ± 5.21 (11.33–32.82)		
Serum creatinine (μmol/L)	905.04 ± 186.41 (491–1196)		

Note: Data are mean ± standard deviation; M, male; F, female; PD, peritoneal dialysis; HD, hemodialysis

^a^Chi square test

^b^Independent samples t-test

**Fig. 1:**
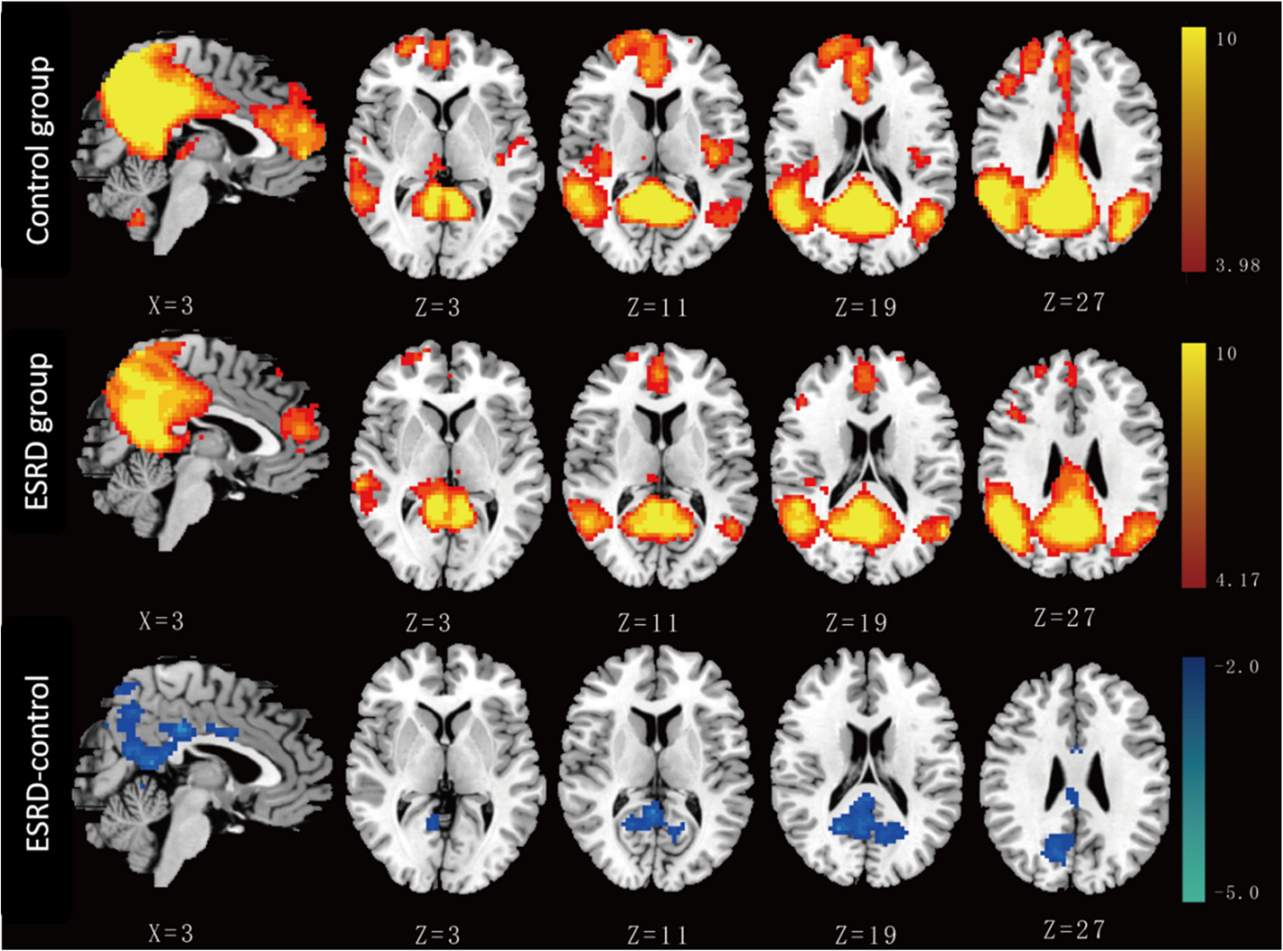
Group comparison based on component 3. The IC map of component 3 in the control group predominantly consisted of the PCC, precuneus, bilateral inferior parietal and MPFC (*P* < 0.001, AlphaSim corrected). Similar functional connectivity but with somewhat disrupted intrinsic connectivity in several DMN areas was found in the ESRD group (P < 0.001, AlphaSim corrected). The ESRD group showed significantly decreased connectivity in the PCC and the adjacent precuneus (*P* < 0.05, AlphaSim corrected, cluster size≥428 voxels) compared with the control group

**Fig. 2:**
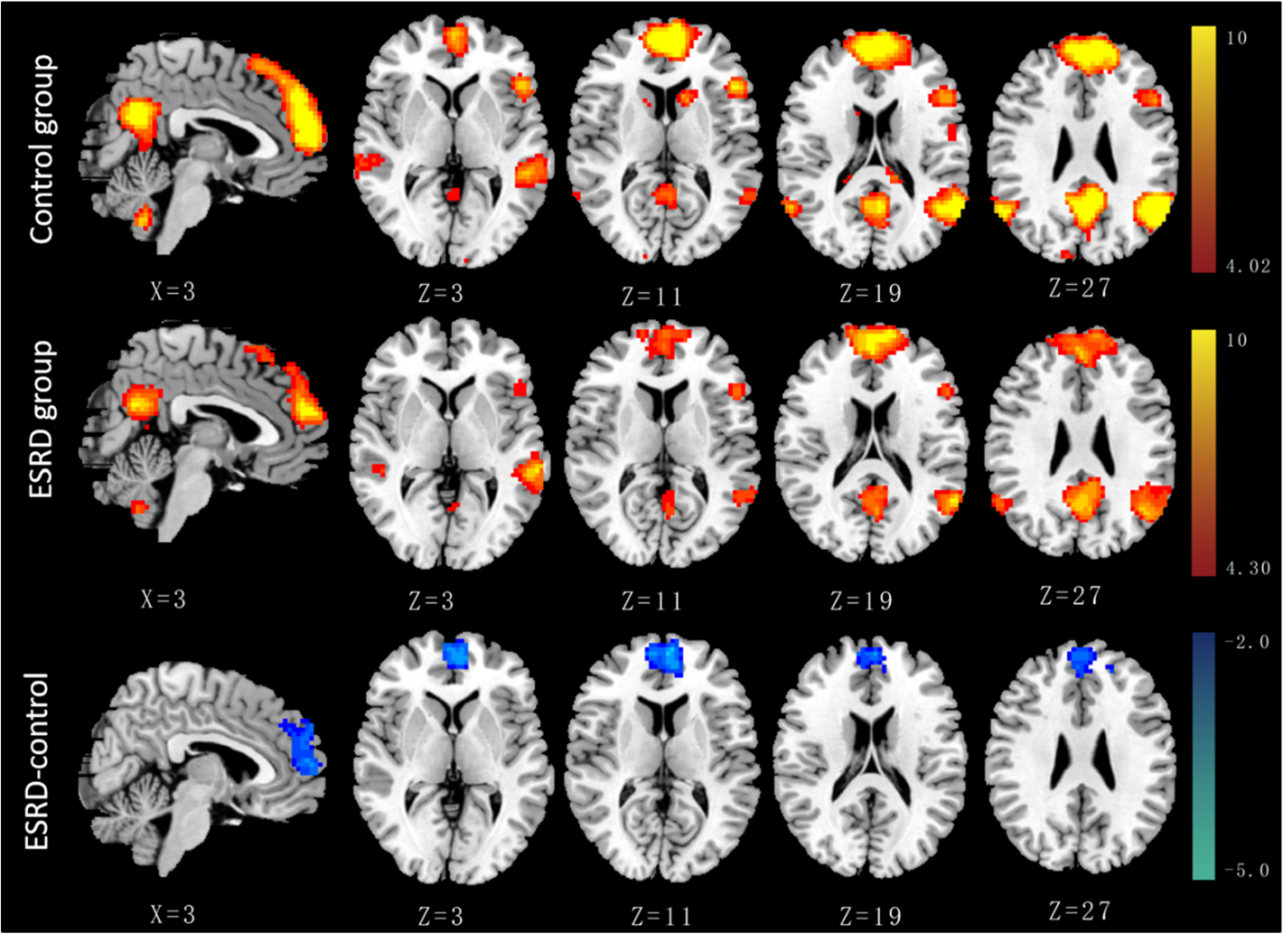
Group comparison based on component 15. The IC map of component 15 in the control group predominantly consisted of the MPFC, PCC and bilateral inferior parietal (P < 0.001, AlphaSim corrected). Similar functional connectivity but with somewhat disrupted intrinsic connectivity in several DMN areas was found in the ESRD group (P < 0.001, AlphaSim corrected). The ESRD group showed significantly decreased connectivity in the MPFC (P < 0.05, AlphaSim corrected, cluster size≥325 voxels) compared with the control group

Group comparisons of FC of the DMN are graphically shown in Fig. 1 (component 3) and Fig. 2 (component 15). For the posterior-DMN (component 3), the ESRD group showed significantly decreased FC in the PCC and precuneus, with significant clusters located in Brodmann’s Area 7 (MNI [x, y, z] coordinates of 6, 9, 33; cluster size of 996; highest z score, 4.13). For the anterior-DMN (component 15), ESRD-associated reduction of FC was observed in the MPFC, with significant clusters located in Brodmann’s Area 9 (MNI [x, y, z] coordinates of 6, 60, 9; cluster size of 636; highest z score, 5.32).

**Fig. 3:**
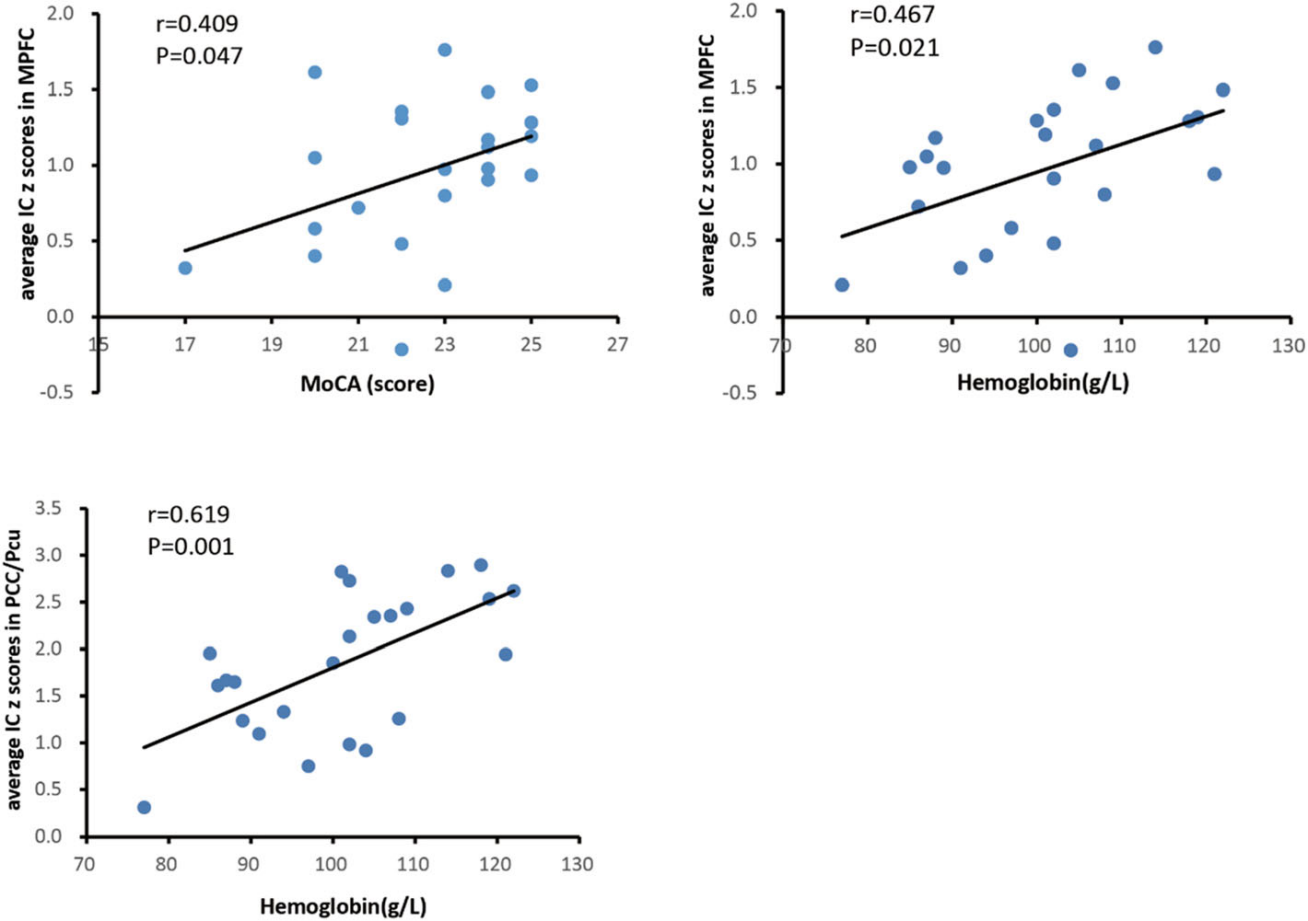
Associations of average IC z score in the MPFC with MoCA score and hemoglobin levels, and of average IC z score in PCC/Pcu with hemoglobin levels. Average IC z score in the MPFC showed positive associations with MoCA score (r = 0.409, *P* = 0.047) and hemoglobin levels (r = 0.467, *P* = 0.021). Average IC z score in the PCC/Pcu was positively correlated with hemoglobin levels (r = 0.619, *P* = 0.001)

Correlation analysis revealed that average IC z score in the MPFC was positively correlated with MoCA score (r = 0.409, *P* = 0.047) and hemoglobin levels (r = 0.467, *P* = 0.021) (Fig. 3). Meanwhile, average IC z score in PCC/Pcu had a positive correlation with hemoglobin levels (r = 0.619, *P* = 0.001) as well (Fig. 3).

**Fig. 4:**
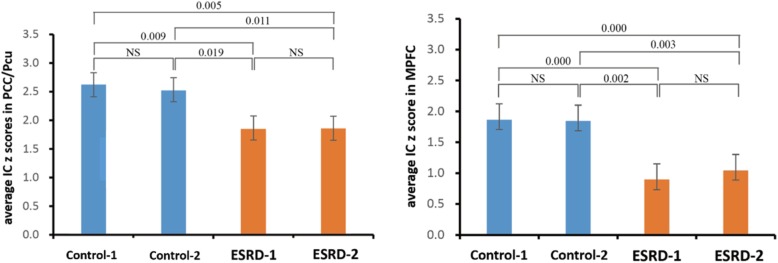
Bar graphs of result reproducibility for evaluating average IC z score of PCC/Pcu and the MPFC in various subgroups (control-1, control-2, ESRD-1 and ESRD-2). NS, no significance

Reproducibility data for evaluating average IC z scores of PCC/Pcu and the MPFC among subgroups are shown on Fig. 4. There were no significant differences in PCC/Pcu or MPFC results between control-1 and control-2, as well as between ESRD-1 and ESRD-2. Meanwhile, differences between control (control-1, control-2) and ESRD (ESRD-1, ESRD-2) subgroups in PCC/Pcu and MPFC results were all very significant. This indicated that the present results had favorable repeatability.

## Discussion

This study demonstrated that impairment of DMN functional connectivity in individuals with ESRD and MCI at rsfMRI. Individuals with ESRD and MCI showed decreased functional connectivity in the PCC, precuneus and MPFC regions. Furthermore, functional connectivity reduction in the MPFC was positively correlated with MoCA score and hemoglobin levels in individuals with ESRD and MCI. Functional connectivity decreases in the PCC and precuneus were positively correlated with hemoglobin levels as well. The present findings complemented previous studies [5, 27, 28] suggesting that metabolic and structural abnormalities are associated with ESRD.

In this study, DMN regions with impaired functional connectivity in individuals with ESRD and MCI, which comprised the anterior hub (MPFC) and posterior hub (PCC) of the DMN, were different from those of MCI caused by degeneration or small vessel disease (SVD). DMN changes in individuals with degeneration are mainly located in the posterior-DMN, especially PCC/Pcu and the medial temporal lobe (MTL) [29]. Deactivation in PCC/Pcu and the MTL is mainly considered a consequence of amyloid accumulation and tau pathology [30]. DMN changes in individuals with SVD are mainly found in the anterior-DMN, mainly the MPFC [31]; the possible mechanism is that patients with SVD have disconnected white matter tracts in the frontal region [31]. The mechanism of impaired FC in individuals with ESRD is likely a combined action of multiple factors, including creatinine and urea accumulation, SVD associated with kidney failure, and dialysis. Posterior leukoencephalopathy might be a promoting factor. Indeed, posterior leukoencephalopathy is not uncommon in ESRD patients undergoing dialysis [32], and the PCC as the posterior DMN hub is often involved in the complications of impaired cerebrovascular autoregulation, endothelial injury and elevated plasma concentrations of natriuretic peptides. Brain microvascular endothelial dysfunction induced by uremic toxins can directly result in SVD; dialysis may bring about the decrease of plasma osmotic pressure, and then induce brain edema; posterior leukoencephalopathy can cause nerve cell dysfunction and even apoptosis, these factors may eventually lead to widespread DMN alteration mainly in the MPFC and PCC/Pcu. These changes of DMN on rsfMRI can be used as imaging markers for the diagnosis of ESRD patients accompanied with MCI in its early stage.

We also found that functional connectivity decrease in the MPFC had a positive correlation with MoCA score, while functional connectivity reductions in the MPFC and PCC/Pcu were positively correlated with hemoglobin levels. MPFC is involved in cognitive processing related to self-awareness, episodic memory, and interactive modulation between internal brain activities and external tasks [33]. Impaired cognitive function might be attributed to functional connectivity alteration in the MPFC. Meanwhile, no significant correlation was found between FC in the MPFC and the MMSE score, which might be due to the weak sensibility of MMSE in evaluating cognitive impairment [14]. Positive associations of decreased functional connectivity in the MPFC and PCC/Pcu with hemoglobin levels were also observed. Previous studies have shown that anemia is associated with cognitive dysfunction in patients with ESRD [34]. This indicates that ESRD patients with MCI may benefit from enhanced hemoglobin levels. In a similar study, Ni et al. [13] also reported that reduced functional connectivity of the DMN is negatively correlated with serum creatinine levels, while we did not observe a significant correlation between FC and serum creatinine levels. We speculate that this discrepancy could be due to dialysis. Indeed, all patients in this study underwent dialysis; thus, serum creatinine levels were well managed. In contrast, participants in Ni et al. were not all being treated by dialysis.

The limitations of this study should be mentioned. First, the sample size was relatively small, which may have affected the statistical power. Secondly, we did not distinguish between various dialysis modalities; serum potassium, hemoglobin, serum albumin, and brain natriuretic peptide levels differed between the HD and PD groups, which might lead to varying degrees of FC impairment in DMN regions. Finally, due to the cross-sectional design, it was impossible to examine the dynamic changes of functional connectivity with ESRD progression, or even FC differences before and after dialysis. Therefore, a longitudinal study is warranted.

## Conclusion

Aiming to improve previous studies focusing on DMN alterations associated with ESRD, we controlled confounding factors better with elaborate grouping and more rigorous exclusion criteria. The present findings indicated that FC in the DMN is impaired in individuals with ESRD and MCI, and such FC changes are correlated with MoCA score and hematocrit levels. These results complemented and/or extended previous reports with additional insights into potential neural underpinnings of asymptomatic ESRD combined with MCI.

## Data Availability

The datasets used during the current study are available from the corresponding author on reasonable request.

## Abbreviations

CDR: Clinical Dementia Rating scale
CKD: Chronic kidney disease
DMN: Default-mode network
ESRD: End-stage renal disease
FC: Functional connectivity
HD: Hemodialysis
ICA: Independent component analysis
MCI: Mild cognitive impairment
MMSE: Mini-Mental State Examination
MoCA: Montreal Cognitive Assessment
MPFC: Medial prefrontal cortex
MTL: Medial temporal lobe
PCC: Posterior cingulate cortex
Pcu: Precuneus
PD: Peritoneal dialysis
rsfMRI: Resting state functional magnetic resonance imaging
SVD: Small vessel disease
